# Glucose prediction by analysis of exhaled metabolites – a systematic review

**DOI:** 10.1186/1471-2253-14-46

**Published:** 2014-06-17

**Authors:** Jan Hendrik Leopold, Roosmarijn TM van Hooijdonk, Peter J Sterk, Ameen Abu-Hanna, Marcus J Schultz, Lieuwe DJ Bos

**Affiliations:** 1 Department of Intensive Care, Academic Medical Center, Meibergdreef 9, 1105 AZ Amsterdam, The Netherlands; 2 Department of Medical Informatics, Academic Medical Center, Meibergdreef 9, 1105 AZ Amsterdam, The Netherlands; 3 Department of Respiratory Medicine, Academic Medical Center, Meibergdreef 9, 1105 AZ Amsterdam, The Netherlands

**Keywords:** Glucose, Monitoring, Volatile organic compound, Breath

## Abstract

**Background:**

In critically ill patients, glucose control with insulin mandates time– and blood–consuming glucose monitoring. Blood glucose level fluctuations are accompanied by metabolomic changes that alter the composition of volatile organic compounds (VOC), which are detectable in exhaled breath. This review systematically summarizes the available data on the ability of changes in VOC composition to predict blood glucose levels and changes in blood glucose levels.

**Methods:**

A systematic search was performed in PubMed. Studies were included when an association between blood glucose levels and VOCs in exhaled air was investigated, using a technique that allows for separation, quantification and identification of individual VOCs. Only studies on humans were included.

**Results:**

Nine studies were included out of 1041 identified in the search. Authors of seven studies observed a significant correlation between blood glucose levels and selected VOCs in exhaled air. Authors of two studies did not observe a strong correlation. Blood glucose levels were associated with the following VOCs: ketone bodies (e.g., acetone), VOCs produced by gut flora (e.g., ethanol, methanol, and propane), exogenous compounds (e.g., ethyl benzene, o–xylene, and m/p–xylene) and markers of oxidative stress (e.g., methyl nitrate, 2–pentyl nitrate, and CO).

**Conclusion:**

There is a relation between blood glucose levels and VOC composition in exhaled air. These results warrant clinical validation of exhaled breath analysis to monitor blood glucose levels.

## Background

Many, if not all, critically ill patients are treated with insulin at some point during their stay in the intensive care unit (ICU) [1]. Intensive monitoring of the blood glucose level is a prerequisite for both efficient and safe insulin titration in these patients [2]. Current practice in the ICU holds that glucose levels are monitored manually through intermittent measurements of the blood glucose level in central laboratories or using laboratory–based blood gas analyzers and/or glucose strips at the bedside [3]. Intermittent manual glucose monitoring however, is expensive and time and blood consuming [4]. Moreover, intermittent glucose monitoring lacks the ability to detect temporal trends, potentially causing dangerous insulin titration errors in critically ill patients [5].

Glucose is a central molecule in metabolism [6,7]. Indeed, metabolic pathways are activated to maintain normoglycemia when the concentration of glucose changes [6,8]. Changes in the activity of these pathways could result in changes in production of volatile metabolites. These so–called volatile organic compounds (VOCs) can be detected in exhaled breath [9].

We hypothesize that there is an association between VOCs in exhaled breath and blood glucose levels. Previous excellent reviews focused on the correlation between glucose and exhaled breath condensate (thus soluble markers) [10] in diabetes [11,12], but none compared all available literature or discussed the implications for the ICU population. The specific aim of this systematic review is to provide an overview of the available data on the association breath VOCs and blood glucose levels and to discuss techniques for VOC detection.

## Methods

This systematic review was done according to standard methodology [13,14]. Medline was searched through Pubmed using the following search terms: (“Blood Glucose”[Mesh] OR “Glucose”[MeSH Terms] OR glucose[tiab]) AND (“Exhalat*” [MeSH Terms] OR “Volatile Organic Compounds” [Mesh] OR exhal* [tiab] OR Volatile Organic Compound* [tiab] OR Volatile Compound* [tiab] OR electronic nose [tiab] OR breath [tiab]. The search was conducted on the 3rd of January 2014. No limits were used for year of publication and language. Only human studies were included, with no restriction on subject health, age, gender or study setting.

Two independent researchers (JHL, LDB) selected articles for full–text assessment when the title and abstract suggested investigating the use of exhaled breath to measure or estimate blood glucose levels. Articles were only included if an association between blood glucose levels and VOCs in exhaled air was investigated. Also, VOC compositions of exhaled air had to be measured by an analytical technique that allows for separation, quantification and identification of individual VOCs, including gas chromatography and mass spectrometry (GC–MS), ion mobility mass spectroscopy (IMS), ion_−_molecule reaction mass spectrometry (IMR–MS), proton transfer reaction (time of flight) mass spectrometry (PTR(−TOF)-MS) and/or selected ion flow tube mass spectrometry (SIFT–MS).

Data from included studies were extracted and methodological quality was assessed independently by two researchers (JHL, LDB) using the QUADAS–2 tool for quality assessment [15]. The tool was adapted to be more relevant to the included literature. Disagreement between the two reviewers on inclusion of studies was resolved by consensus. The adjusted version of QUADAS–2 is presented in Additional file 1. Extracted data included: 1) characteristics of the study (design, year of publication and country of study conduction); 2) characteristics of the study population (including age, sex distribution and health status); 3) characteristics of the index test (including technique and included VOCs); 4) characteristics of the reference standard (blood glucose); 5) characteristics of the outcome (including main results and correlation coefficient between exhaled breath and glucose); 6) statistical validation technique used.

## Results

### Search results

**Figure 1:**
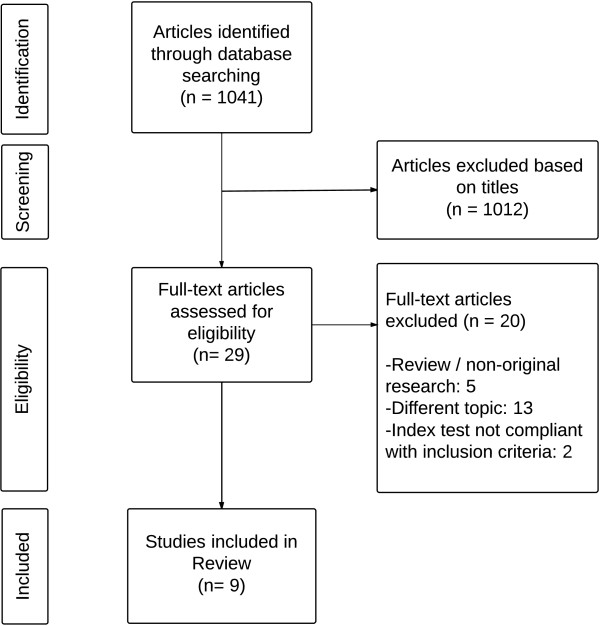
Flow diagram.

The literature search in Pubmed yielded 1041 titles (Figure 1). After reading titles and abstracts, 1012 articles were excluded because the topic was outside of the scope of this review and 29 articles were retained for full–text assessment. After the exclusion of 20 papers (5 reviews/non-original studies, 13 on unrelated topics, 2 index test not compliant with inclusion criteria), 9 articles were included in the analysis. Characteristics of selected articles are presented in Table 1. Five studies included healthy non-diabetic subjects, two studies included Type 1 Diabetes Mellitus (T1DM) subjects, one study included Type 2 Diabetes Mellitus (T2DM) subjects and one study included both healthy and T1DM subjects.

**Table 1:** Characteristics of included studies

**First Author**	**Setting**	**Patients**	**Age**	**Sex distribution**	**Index test**	**Reference standard**	**Main Results**	**Mean correlation coefficient**
Righettoni [17]	Healthy subjects sampled after overnight fast and after lunch.	8	22-55 years	7 male, 1 female	PTR-TOF-MS: Acetone, ethanol, methanol, isoprene Nano sensing films: Breath acetone	Finger prick measurement with Bayer Contour Blood Glucose Meter	After overnight fast a high correlation between sensors and glucose, and acetone, ethanol, methanol and glucose was found. These high correlations were not found after lunch.	Morning: PTR-TOF-MS: Acetone:0.98 Ethanol:0.9 Methanol: .93 Isoprene:0.00 Nano sensing films: 0.96 Afternoon: PTR-TOF-MS: Acetone:-0.08 Ethanol:0.11 Methanol:-0.16 Isoprene:-0.40 Nano sensing films: −0.02
Storer [18]	T2DM subjects not asked to fast but to refrain from eating. Cross-sectional study	38, T2DM	32-76 years, median age 62	13 male, 25 female	SIFT-MS: Acetone	Finger prick measurement with Abbot Optium Xceed	No strong correlation found between blood glucose and breath acetone. Breath acetone was found to be significantly higher in men.	r = 0.003
Minh [22]	Clamp study. Overnight fast. T1DM subjects were asked not to take long acting insulin.	25 (17 healthy, 8 T1DM)	Healthy: 28 ± 1 years T1DM: 25,8 ± 1,7 years	11 male, 14 female	GCMS: Group A (Ethanol, acetone, methyl nitrate, ethyl-benzene) Group B (2-pentyl nitrate, propane, methanol, ethanol) Room samples collected.	IV catheters in antecubital veins; Beckman Glucose analyzer II	Group A: healthy, mean r of 0.836, T1DM, mean r of 0.950. B: healthy, mean r of 0.829, T1DM, mean r of 0.920.	Healthy: r = 0.8325 T1DM: r = 0.935
Turner [21]	Clamp study. Overnight fast. T1DM subjects	8, T1DM	28 ± 3 years		SIFT-MS: Acetone	IV distal catheter in hand. Hand warmed to arterialize the sample. YSI.	No strong correlation at baseline. Linear correlation between acetone and blood glucose values. Breath acetone decreased when blood glucose decreased. In healthy volunteers the opposite was seen: Low blood glucose values yield high acetone values.	r = 0.816(0.598-0.940)
Lee [20]	Clamp study. Healthy subjects admitted to lab after overnight fast.	10	26 ± 4 years	5 male, 5 female	GCMS: Ethanol, Acetone, Methyl nitrate, ethylbenzene, o-oxylene, m/p-xylene. Room samples collected.	IV catheters in antecubital veins; Beckman Glucose analyzer II	Best 4 gas model: Ethanol, acetone, methyl nitrate, ethyl benzene (mean r of 0.913(0.698-0.977)) 9 samples per patient	r = 0.913 (0.698-0.977)
Fritsch [19]	OGTT. Healthy volunteers admitted after 10 hours fast.	6	24-32 years	5 male, 1 female	Electrochemical analyzer, laser spectrometer, and breath hydrogen: Carbon monoxide measured with Micro smokerlyzer.	Finger prick measurement, Accu check Aviva.	No strong correlation between glucose and carbon monoxide	None
Novak [39]	Clamp study. T1DM subjects admitted after eating light breakfast. Patients on insulin followed normal regimen.	10, T1DM	13,8 ± 0,5 years	7 male, 3 female	GCMS: Methyl nitrate Room samples collected.	IV lines in arms, Blood samples every 30 min. Beckman glucose analyzer II	Methyl nitrate had strongest correlation with blood glucose levels. Correlation increased with 30-minute lag time. Ethanol and Acetone DID NOT correlate with glucose	One subject mentioned, r = 0.99
Galassetti [32]	OGTT. Healthy subjects admitted to research center in morning after overnight fast.	10	27,4 ± 3,1	5 male, 5 female	GCMS: Ethanol and acetone. Room samples collected.	IV catheter. Determined with a quantitative enzymatic measurement.	Multiple linear regression analysis with ethanol and acetone gave an average r of 0.70.	r = 0.700
Paredi [27]	OGTT in 5 patients, CO and glucose measured. Only CO measured in larger cohort	5	33 ± 4 years	3 male, 2 female	Micro smokerlyzer: Carbon monoxide	Finger prick measurement, Reflolux S.	The maximal glucose increase was associated with a significant increase in exhaled CO concentration. Both parameters returned to the baseline at 40 min after glucose administration.	Unknown

Results of the quality assessment using the QUADAS–2-tool are presented in Table 2. The risk of bias was considered high for all studies; none of the studies used a random sample of patients, all using a pre-specified target group such as T1DM or T2DM patients. The use of blood gas measurements or central lab measurements was considered to be the correct reference standard [16]. The adequate reference standard was used in four studies. Four studies used finger prick measurement, which increases the possibility of incorrect insulin titration in clinical practice [16]. Comparing these measurements to exhaled breath could lead to biased results. However, none of these studies were excluded from our review.

**Table 2:** Results of QUADAS-2 tool

**Study**	**Risk of Bias**				**Applicability Concerns**			
	**Patient selection**	**Index test**	**Reference standard**	**Flow and timing**	**Patient selection**	**Index test**	**Reference standard**	**Comments**
Righettoni (2013) [17]	?	?	×	✓	✓	✓	✓	Single measurements in morning and in afternoon make prediction of trend impossible. Possible verification bias because of incorrect reference standard.
Storer (2011) [18]	?	×	×	✓	×	×	✓	Single measurement makes prediction of trend impossible. Test review bias because reference standard is used for index test. Possible verification bias because of incorrect reference standard
Minh (2011) [22]	?	✓	✓	✓	✓	✓	✓	Clamp study design possibly lowers clinical relevance because of lack of generalizability. Test review bias because reference standard is used for index test.
Turner (2009) [21]	?	×	✓	✓	×	×	✓	Small sample size. Clamp study design possibly lowers clinical relevance because of lack of generalizability. Test review bias because reference standard is used for index test.
Lee (2009) [20]	?	×	✓	✓	✓	✓	✓	Small sample size. Clamp study design possibly lowers clinical relevance because of lack of generalizability. Test review bias because reference standard is used for index test.
Fritsch (2008) [19]	?	✓	×	✓	✓	×	✓	Small sample size. OGTT study design possibly lowers clinical relevance because of lack of generalizability. Test review bias because reference standard is used for index test. Possible verification bias because of incorrect reference standard.
Novak (2007) [39]	?	×	✓	✓	×	✓	✓	Clamp study design possibly lowers clinical relevance because of lack of generalizability. Test review bias because reference standard is used for index test. Possible reporting error, results of only one subject mentioned in detail.
Galassetti (2005) [32]	?	×	?	✓	✓	✓	✓	Small sample size. OGTT study design possibly lowers clinical relevance because of lack of generalizability. Test review bias because reference standard is used for index test. Possible verification bias because of incorrect reference standard
Paredi(1999) [27]	?	✓	×	✓	✓	×	✓	Small sample size. OGTT study design possibly lowers clinical relevance because of lack of generalizability. Possible verification bias because of incorrect reference standard.

### Point correlation

Authors of seven out of nine studies found a strong correlation between one or more metabolites in exhaled breath and blood glucose levels, with a mean linear regression coefficient of 0.82 [range: 0.08-0.98] (Table 1). However, none of the included studies validated their results internally (e.g. cross-validation) or externally (e.g. in an separate validation cohort). A total of ten metabolites have been reported to correlate with blood glucose levels, including exhaled acetone, VOCs produced by gut flora (ethanol, methanol, and propane), exogenous compounds (Ethyl benzene, o-xylene, and m/p-xylene) and VOCs that reflect oxidative status (methyl nitrate, 2-pentyl nitrate, and carbon monoxide (CO)) (Table 3). Authors of two studies did not observe a strong correlation. The first one of those did not find a significant correlation between a *single* measurement of breath acetone and blood glucose in T2DM subjects. Authors of the second study were unable to demonstrate a strong correlation between glucose levels and exhaled CO in healthy subjects. Researchers in one of the studies that did show a strong correlation between breath metabolites and glucose levels, only observed this after overnight fast, showing a weak correlation after consuming a meal [17].

**Table 3:** VOCs found to correlate with glucose levels

**VOC**	**Mechanism(s)**	**Pathway(s)**
2-pentyl nitrate [22]	Generated through pathways involving organic peroxy radical (RO2▪) with NO or NO2. Could be modulated by acute changes in systematic oxidative status [22].	
Acetone [20-22,32]	Derived from acetoacetate and is produced by synthesis and degradation of ketone bodies and is therefore related to blood glucose levels [32].	Glycolysis/Pyruvate metabolism
Cabon monoxide [27]	Possibly due to activation of HO by glucose, and the positive modulation of CO non insulin secretion [27].	
Ethanol [20,22,32]	Not produced by mammalian cells. Likely due to alcoholic fermentation of glucose by gut bacteria and yeast [32].	Glycolysis/Gluconeogenesis
Ethyl benzene [20,22]	Inhaled and partly metabolized by liver, then exhaled at lower concentration. Rapid-onset hyperglycemia likely suppressed hepatic metabolism causing peaks in exhaled air [20].	
M/P-xylene [20]	Inhaled and partly metabolized by liver, then exhaled at lower concentration. Rapid-onset hyperglycemia likely suppressed hepatic metabolism causing peaks in exhaled air [20].	
Methanol [22]	Reflects gut flora activity and therefore responsive to glycemic fluctuations [22].	
Methyl nitrate [22,39]	A small fraction of superoxide ion (O2▪−), a byproduct of oxidative reactions, reacts with nitric oxide which in turn can react with methanol to eventually form an isomer of Methyl nitrate [39].	
O-xylene [20]	Inhaled and partly metabolized by liver, then exhaled at lower concentration. Rapid-onset hyperglycemia likely suppressed hepatic metabolism causing peaks in exhaled air [20].	
Propane [22]	Reflects gut flora activity and therefore responsive to glycemic fluctuations [22].	N-4 fatty acid Peroxidation Protein oxidation

### Temporal association

Researchers in seven out of nine studies performed multiple measurements with an interval ranging from 2.5 to 40 minutes. Two studies had a cross-sectional design and only performed a single measurement, or two unpaired measurements. None of the authors of the included studies reported on the possibility of predicting glucose trend.

## Discussion

This systematic review identified nine studies that investigated the ability of exhaled breath to measure or estimate blood glucose levels. A significant correlation between VOCs in exhaled breath and blood glucose levels was found in seven studies. These results indicate that there is an association between the two, although not all studies are consistent. Researchers in one of these seven studies only found a strong correlation after overnight fasting of the subjects and were unable to replicate the results after a meal [17]. Authors of two negative studies did not find a strong correlation, possibly due to a different study design [18] and the VOC (e.g. CO) that was studied [19]. Authors of the study that included subjects with T2DM did not show a significant correlation between exhaled VOCs and blood glucose levels. This study also had a different (cross-sectional) design. The analytical technique used for VOC detection did not modify the reported correlation. None of the studies monitored breath continuously. Also the glucose trend, thus the temporal association between glucose and exhaled VOCs, was not explicitly investigated. However, the data from three longitudinal studies [20-22] suggest that trends in glucose levels could possibly be monitored using exhaled breath when measurements are taken more frequently.

### Index tests: exhaled breath analysis

A significant correlation between metabolites in exhaled breath and blood glucose levels was found using GC-MS, SIFT-MS, PTR-TOF-MS, a nano-sensing film-based sensor, and an electrochemical analyzer as analytical method. GC-MS is considered to be the gold standard for VOC detection and has shown to have a high sensitivity to identify single VOCs [23]. Therefore, GC-MS is suitable to accurately quantify a number of different VOCs in a cross-sectional study. However, the time-consuming nature of the technique limits use of the device for real-time and continuous measurements, which hampers clinical application. Other analytical techniques such as SIFT-MS [22,23] and PTR-TOF-MS [23-25] can also identify single VOCs and can be used for real-time continuous measurements. Disadvantages include possible selection bias [26] and the limitation to the concentration range that can be detected [25].

The electrochemical analyzers used in selected studies are two different Smokerlyzer Micro (Bedfont, UK) devices. These devices measure the amount of CO in exhaled breath. However, there is a cross sensitivity to hydrogen [19]. While a correlation between exhaled CO and glucose levels was found by the researchers of one study [27], researchers of another study [19] could not reproduce these findings. Contrasting results may be due to the high cross sensitivity to hydrogen in the electrochemical analyzer used previously [27], which was less apparent using a newer device [19]. This exemplifies the importance of an adequate analytical technique that suits the aim of the study.

An important limitation of the techniques used in included studies is that none of them was used to continuously monitor exhaled breath. Continuous analysis of the exhaled breath was previously described by means of IMR-MS [28], PTR-MS [24], and electronic nose [29,30]. After a training phase, electronic noses learn to recognize specific disease states and can therefore be used for classification. The devices cannot identify and quantify single VOCs, but they do give a rapid, bedside diagnosis, which, from a clinical perspective, renders this device attractive. The electronic nose has been used to discriminate between patients with and without diabetes [31]. One could postulate that the ability to diagnose diabetes is partly due to the metabolomic alterations because of higher blood glucose levels in diabetic patients. Therefore, electronic nose analysis may complement mass-spectrometry based techniques for the monitoring of blood glucose levels in clinical practice, providing signals based on probabilistic training and validation. Alternatively to semi-selective recognition, nanosensors also rely on specific recognition of certain VOCs [30]. In one study, an acetone-selective nanomaterial-based sensor was used alongside PTR-TOF-MS and showed a strong correlation between acetone, glucose and the sensor [17]. Small size of devices using nanomaterial-based sensors as compared to spectrometry-based methods facilitates clinical application.

### VOCs associated with blood glucose levels

Mechanisms related to the association between VOCs and glucose levels can be found in Table 3. Acetone appeared to be associated with blood glucose levels [17,20-22,32]. As a result of increased synthesis of acetone and degradation of ketone bodies, acetone is expected to be higher in diabetics [33]. On the other hand, healthy humans only have elevated levels of ketone bodies when fasting or exercising [11]. Therefore, it is more likely to find a correlation between exhaled acetone and glucose levels after fasting compared to finding a correlation after lunch [17]. Acetone possibly is a good marker for glucose levels in ICU-patients. However, the large variation in breath acetone levels between subjects [18,34-36] may result in low accuracy when using acetone cross-sectionally.

VOCs such as ethanol [17,20,22,32], propane [22] and methanol [17,22] are likely to reflect gut flora activity, since the metabolism of gut bacteria is responsive to glycemic fluctuations [22,32]. However, we cannot exclude that other biochemical pathways also contribute to the production of these compounds. In critically ill patients on the ICU, the quantity and composition of the gut microbiome are changing over time and therefore the amount of VOCs they produce may not be stable [37] Therefore, these markers are less likely to predict glucose levels in ICU-patients.

Ethyl benzene [20,22], o-xylene [20] and m/p-xylene [20] are gasses that are inhaled, partially metabolized by the liver and subsequently exhaled at lower concentrations [20]. Rapid-onset hyperglycemia likely suppresses hepatic metabolism, thus causing peak concentrations of these compounds in exhaled air. Recent evidence suggests that cyclic hydrocarbons such as ethyl benzene and xylene are emitted by the ventilator and tubing [38]. Given that exhaled air is readily accessible for measurements in mechanically ventilated ICU patients, use of exhaled air for the prediction of glucose levels is therefore plausible.

An isomer of methyl nitrate [22,39] is formed when methanol reacts with nitric oxide, which in turn reacts with superoxide ion (O_2_^-^), a by-product of oxidative reactions [39]. Furthermore, 2-pentyl nitrate [22] is generated through pathways involving organic peroxy radical (RO_2_) and NO or NO_2_. This could be modulated by acute changes in systematic oxidative status [22]. Changes in CO [27] in exhaled breath are possibly related to oxidative stress. When glucose levels rise, particularly in diabetic patients, this can lead to oxidative stress. As a protective response, heme oxygenase is activated, leading to the positive modulation of CO on insulin secretion [27]. For critically ill patients on the ICU however, markers of oxidative stress will be non-specific for high blood glucose, as they increase with any form of oxidative stress such as sepsis, high inspired-oxygen fraction and acute respiratory distress syndrome [40].

### Study design

The observed correlation between blood glucose levels and exhaled VOCs may be due to the inclusion of T2DM patients and/or a cross-sectional study design. First, T2DM influences the responsiveness of the body to changes in blood glucose levels [41]. This is typically characterized by insulin resistance but may also influence the formation of ketone bodies and the induction of liver enzymes. Second, breath acetone levels tend to differ between T1DM, T2DM, and healthy subjects [18,21,35]. Therefore, a decrease in blood glucose levels may not induce the same rise in breath acetone levels with different baseline values and in the context of different co-morbidities. Finally, in line with the previous point, correction for baseline differences between subjects cannot be accomplished with a cross-sectional study design. This is further acknowledged by the fact that the predictive algorithm requires calibration for every subject in several studies [20,22]. Since the relation between exhaled breath metabolites and blood glucose levels shows high inter-person variation, a cross-sectional design may not be ideal for predicting glucose levels using breath metabolites. The possibility of using a single breath maneuver to estimate blood glucose levels thus seems implausible. Future studies may therefore focus on longitudinal measurements in the same subject.

Five included studies used a clamp study design and 2 studies used an oral glucose tolerance test (OGTT). Clamp studies and OGTT result in a more or less predictable course of blood glucose levels. Although a clamp design is ideal for research purposes and enables comparability between studies, clinical practice is often very different and less predictable. The transition of the results of these studies to the clinical setting will be a major challenge for the field of blood glucose estimation by exhaled breath analysis.

### Strengths and limitations

We used a standardized systematic review approach, combining all evidence available. All VOCs that are linked to changes in glucose levels are discussed and their most likely biochemical pathways are described. In addition, we carefully assessed the quality of the included studies.

This systematic review also has an important limitation. The included studies were highly heterogeneous with respect to patient selection, exhaled breath sampling and analysis and blood glucose measurement, limiting the comparability of the studies. Therefore, we decided to describe the results separately. Most of the included studies had a relatively high risk of bias and we found that included studies did not validate their results. Possibly, this is inevitable in the early stages of biomedical research but it hinders strong conclusions. Furthermore, models can possibly be overfit, yielding overoptimistic results. Our search only identified one negative study. Negative studies are often not published leading to publication bias.

None of the studies investigated ICU-patients, while glucose fluctuations are large and frequent in this population [42]. Therefore, we cannot draw firm conclusions on the use of these methods in ICU-patients. We did try to identify potential pitfalls for the implementation of these methods in ICU patients by reviewing the biochemical pathways for the formation of VOCs.

Finally, the use of exhaled breath to monitor glucose trends was not discussed in any of the articles. Monitoring glucose trends (in ICU patients) however, has several potential advantages over using single values. First, trend has a better predictive value compared to single glucose levels; recent trend can be used to predict future levels. In ICU patients, this can lead to improved insulin titration. Second, because outliers can be filtered out, trend is less susceptible to random noise. Third, possible bias (constantly predicting values too high/low) will be constant throughout the trend, having a smaller effect. Potential disadvantages of using glucose trend are possible lag in the signal, and the potential of amplification of errors.

## Conclusion

In conclusion, a significant association between VOCs in exhaled breath and blood glucose levels was found in the majority of studies included in this systematic review. Acetone, carbon monoxide, ethanol, ethyl benzene, M/P-xylene, methanol, O-xylene, and propane were correlated with blood glucose levels. Several potential effect modifiers were identified for ICU-patients. The included studies were performed under highly controlled circumstances, which limit generalizability. Our results warrant clinical validation of exhaled breath analysis for the monitoring of blood glucose levels in critically ill ICU-patients.

## Abbreviations

CO: Carbon monoxide; GC–MS: Gas chromatography and mass spectrometry; ICU: Intensive care unit; IMS: Ion–mobility spectroscopy; OGTT: Oral glucose tolerance test; PTR-(TOF)-MS: Proton transfer reaction (time of flight) mass spectrometry; SIFT-MS: Selected ion flow tube mass spectrometry; T1DM: Type 1 diabetes mellitus; T2DM: Type 2 diabetes mellitus; VOC: Volatile organic compound.

## Competing interests

The authors declare that they have no competing interests.

## Authors’ contributions

JHL designed the review, extracted the data, summarized the findings, and composed the manuscript. RTMvH participated in the study design and contributed to the manuscript. PJS participated in the study design and contributed to the manuscript. AAH participated in the study design and contributed to the manuscript. MJS participated in the study design, contributed to the manuscript and coordinated the team efforts. LDB participated in the study design, extracted the data, summarized the findings and contributed to the manuscript. All authors read and approved the final manuscript.

## Pre-publication history

The pre-publication history for this paper can be accessed here:

http://www.biomedcentral.com/1471-2253/14/46/prepub

## Supplementary Material

Additional file 1 QUADAS-2 - Adapted for systematic review on Glucose Prediction by Analysis of Exhaled Metabolites. Click here for file
